# Quantification of Brain β-Amyloid Load in Parkinson's Disease With Mild Cognitive Impairment: A PET/MRI Study

**DOI:** 10.3389/fneur.2021.760518

**Published:** 2022-03-01

**Authors:** Michela Garon, Luca Weis, Eleonora Fiorenzato, Francesca Pistonesi, Annachiara Cagnin, Alessandra Bertoldo, Mariagiulia Anglani, Diego Cecchin, Angelo Antonini, Roberta Biundo

**Affiliations:** ^1^Parkinson and Movement Disorders Unit, Department of Neuroscience, University of Padua, Padua, Italy; ^2^Department of General Psychology, University of Padua, Padua, Italy; ^3^Department of Neuroscience, University of Padua, Padua, Italy; ^4^Padova Neuroscience Center, University of Padua, Padua, Italy; ^5^Department of Information Engineering, University of Padua, Padua, Italy; ^6^Neuroradiology Unit, Padua University Hospital, Padua, Italy; ^7^Nuclear Medicine Unit, Department of Medicine - DIMED, Padua University Hospital, Padua, Italy; ^8^Study Center for Neurodegeneration, University of Padua, Padua, Italy

**Keywords:** Parkinson's disease, mild cognitive impairment, amyloid-β, atrophy, cognition, executive functions, dementia, PET

## Abstract

**Background:**

Mild cognitive impairment in Parkinson's disease (PD-MCI) is associated with faster cognitive decline and conversion to dementia. There is uncertainty about the role of β-amyloid (Aβ) co-pathology and its contribution to the variability in PD-MCI profile and cognitive progression.

**Objective:**

To study how presence of Aβ affects clinical and cognitive manifestations as well as regional brain volumes in PD-MCI.

**Methods:**

Twenty-five PD-MCI patients underwent simultaneous PET/3T-MRI with [^18^F]flutemetamol and a clinical and neuropsychological examination allowing level II diagnosis. We tested pairwise differences in motor, clinical, and cognitive features with Mann–Whitney U test. We calculated [^18^F]flutemetamol (FMM) standardized uptake value ratios (SUVR) in striatal and cortical ROIs, and we performed a univariate linear regression analysis between the affected cognitive domains and the mean SUVR. Finally, we investigated differences in cortical and subcortical brain regional volumes with magnetic resonance imaging (MRI).

**Results:**

There were 8 Aβ+ and 17 Aβ- PD-MCI. They did not differ for age, disease duration, clinical, motor, behavioral, and global cognition scores. PD-MCI-Aβ+ showed worse performance in the overall executive domain (*p* = 0.037). Subcortical ROIs analysis showed significant Aβ deposition in PD-MCI-Aβ+ patients in the right caudal and rostral middle frontal cortex, in precuneus, in left paracentral and pars triangularis (*p* < 0.0001), and bilaterally in the putamen (*p* = 0.038). Cortical regions with higher amyloid load correlated with worse executive performances (*p* < 0.05). Voxel-based morphometry (VBM) analyses showed no between groups differences.

**Conclusions:**

Presence of cerebral Aβ worsens executive functions, but not motor and global cognitive abilities in PD-MCI, and it is not associated with middle-temporal cortex atrophy. These findings, together with the observation of significant proportion of PD-MCI-Aβ-, suggest that Aβ may not be the main pathogenetic determinant of cognitive deterioration in PD-MCI, but it would rather aggravate deficits in domains vulnerable to Parkinson primary pathology.

## Introduction

Cognitive alterations in Parkinson's disease (PD) are among the most disabling non-motor symptoms, and they impact negatively on patient's and caregiver's quality of life and can be present already in early stages of disease. Parkinson's disease with mild cognitive impairment (PD-MCI) have up to six-fold higher risk to develop dementia (PDD) (1). However, the characteristics and the severity of the cognitive profile as well as the rate of progression to dementia are heterogenous (2, 3). Factors contributing to variability in PD cognitive performance are: (a) presence of specific genetic mutations or variants (4), (b) characteristics of phenotypic manifestations including dominant akinetic rigid form or early occurrence of postural instability and hallucinations, (c) variable expression of synuclein pathology, in particular presence of cortical Lewy bodies, (d) presence of misfolded β-amyloid (Aβ), and in some cases of tau neurofibrillary tangles, which are considered typical Alzheimer hallmarks. Magnetic resonance imaging (MRI) studies in PD-MCI have reported variable structural and functional patterns without clarifying the underlying mechanisms (5–7). Some studies have suggested contribution of Aβ cortical and subcortical depositions to cognitive decline in PD particularly in association with attentive and executive deficits, while others indicated an increased risk of dementia in the late disease stages (8–13). These differences may be related to the heterogeneous methodology adopted including variability in age and sex as well as poorly characterized cognitive diagnosis in relatively small cohorts (11, 14–17). In a recent PET/MRI study (18) in cognitively well-characterized Lewy Body disease (LBD) patients, we observed an integral role of brain amyloidosis in cognitive profile and progression, affecting mainly global cognition (MoCA, MMSE), attentive/executive, and semantic recall abilities. Our study also confirmed the Aβ contribution to cognitive dysfunction in a significant proportion of our Lewy body dementia subjects, although half of the demented patients were Aβ-. However, we did not explore whether and at which extent presence of amyloid deposition contributes specifically to MCI status in PD patients. Considering MCI established heterogeneity as well as its greater vulnerability to dementia, the purpose of this analysis is to investigate whether amyloidosis distinguishes a specific PD-MCI profile across the various and heterogeneous patterns and if so its contribution to dementia development. Hence, in the current study, we expanded previous analysis of PET/MRI data and focused specifically on the quantification of [^18^F]flutemetamol (FMM) deposition in the PD-MCI cohort, and in their related cognitive, clinical, and brain structural correlates.

## Methods

### Participants

Data of 25 PD-MCI were analyzed from the cohort recruited in the context of a previously published study (18). Patients were recruited at the Parkinson's Disease and Movement Disorders Unit of Neurology Clinic in Padua from 2016 to 2020. Parkinson's disease diagnosis was based on the most recent MDS clinical diagnostic criteria (19), confirmed by abnormal DaTscan SPECT imaging. Exclusion criteria included deep brain stimulation, atypical Parkinsonian disorders, severe psychiatric or neurological comorbidity, presence of pathogenic genetic mutations, and clinically relevant cerebrovascular disease on MRI. All participants underwent a complete neuropsychological evaluation, simultaneous PET/MRI with FMM, and a genetic assessment. A customized genetic panel (more than 90 genes associated to Movement Disorders) was used to analyze patients' DNA, and only individuals without genetic mutations were further included in this study. Regarding genetic variability as a possible confounding factor, we excluded from this study subjects carrying known PD genetic mutations and variants, but we did not screen for apolipoprotein E (APOE) ε4. In particular, mutations of the glucocerebrocidase (GBA) gene have been associated with more rapid cognitive decline in PD-MCI, with subsequent α-synuclein deposition enhancement as well as effects in proteins implicated in dopamine production, metabolism, and signaling (20).

The study was approved by the Ethic Committee of the University of Padua (4340/AO/17). All patients gave written informed consent according to the Declaration of Helsinki.

### Clinical and Neuropsychological Examination

Demographic and clinical characteristics were collected by expert neurologists (AA, ACC). The severity of extrapyramidal symptoms was assessed with the motor Unified Parkinson's Disease Rating Scale (21) as well as with the Hoehn and Yahr score “on” medication. Levodopa Equivalent Daily Dose and Dopamine Agonist Equivalent Daily Dose were calculated for each patient (22). Patients' age at disease onset was defined as the age at which they noticed the first motor symptom suggestive of PD. All participants underwent a comprehensive neuropsychological evaluation, in line with the MDS task force level II PD-MCI diagnostic criteria (23) [for further details on cognitive tests adopted, see Fiorenzato et al. (24)]. In all patients the evaluation of functional and instrumental activities of daily living was performed independently of the impairment ascribable to motor or autonomic symptoms. Regarding the behavioral evaluation, the Beck Depression Inventory (BDI-II), Starkstein Apathy Scale, and the State-Trait Anxiety Inventory (STAI-Y1 and Y2) were used to detect the presence of eventual depression, apathy, state and trait anxiety, respectively. Patients were evaluated in “on” medication state. The cognitive tests were administered by trained neuropsychologists, in the morning, on two separate occasions within 3–5 days.

### PET/MRI Acquisition and PET/FMM Images Classification

Parkinson's disease patients in accordance with the amyloid imaging procedure guidelines (25) received an intravenous injection of about 185 MBq FMM (performed manually over 10 s and flushed with 30 ml of saline over about 15 ± 5 s) directly in an integrated 3T PET/MRI system (Biograph mMR; Siemens, Erlangen, Germany). Images were acquired between 0–10 and 90–110 min after injection according to Cecchin et al. (26). Anatomical volumetric data *via* T1-weighted-3D magnetization-prepared rapid acquisition gradient echo sequence (TR 1.900 ms, TE 2.53 ms, slice thickness 1 mm, matrix 256 × 256, FOV 250 mm) were simultaneously acquired. Additionally, a 1 mm-isotropic T2-weighted-3D, and Two-Dimensional Susceptibility-Weighted Imaging, were acquired for clinical evaluation, excluding secondary parkinsonisms, the presence of vascular brain damage, and allowing visual rating scales assessment.

Visual assessment of FMM images is a robust and reliable method for detection of brain neuritic Aβ plaques (25). A binary visual classification was performed by an expert nuclear medicine physician (DC, with both in-person and e-training), blinded to cognitive status and diagnosis, who rated each scan as amyloid-positive (Aβ+) or negative (Aβ-).

For further details about PET reconstruction and PET/FMM images classification procedures see Biundo et al. (18).

### PET Quantification

The PET frames were realigned, averaged, and co-registered with their respective MRI scans with the Freesurfer v7.01 (http://surfer.nmr.mgh.harvard.edu/). Since realignment might bias standardized uptake value ratios (SUVRs) comparison and partial volume correction (PVC) estimation (27), in order to improve the reliability of the realignment process, both T1w3d and PET were first manually z-cropped including medulla and cerebellum and rigidly realigned to the anterior commissure-posterior commissure line (AC-PC line) using the Freeview Freesurfer tool. We performed voxel-based three-compartment PVC to the MRI coregistered PET images using the PETSurfer tool in FreeSurfer (28, 29). Standardized uptake value ratios were computed to address intersubject effects using cerebellar gray matter (GM) as the reference region.

### PET SUVR Subcortical ROIs

Fourteen subcortical striatal and extra striatal regions ROIs (nucleus accumbens, caudate, putamen, amygdala, globus pallidus, thalamus, and hippocampus) were extracted in the native MRI space using Freesurfer v7.01 Desikan/Killiany atlas 11 segmentations (30). Standardized uptake value ratio maps were then projected to the subcortical regions to extract the mean value for each PD patient.

### PET SUVR Cortical Surface-Based Analysis

Vertex-wise general linear model (GLM) (between group comparisons) comparing cerebral cortical SUVR of the PD-MCI Aβ+/– was run using Freesurfer. Standardized uptake value ratio maps of each subject were sampled onto the left and right surfaces *via* the individual subject's surface, and a surface-based smoothing of 8-mm full width at half maximum was applied. Surface areas of significant amyloid load, which survived a cluster-wise Monte Carlo correction for multiple comparisons after running 10,000 permutations, were considered for the following steps.

### PET SUVR Subcortical Analysis

Mean subcortical SUVR values were compared between hemispheres using the Wilcoxon test and tested for Left-Right hemisphere correlation using the non-parametric Spearman test. Subcortical regions with an inter-hemisphere difference *p* > 0.2 and a correlation significance of *p* < 0.001 were averaged, to reduce the degrees of freedom in multiple comparisons testing as well as to increase the statistical power. The obtained values were compared between PD-MCI-Aβ+ and Aβ- with a non-parametric Mann–Whitney U test for amyloid load.

### Voxel Based MRI Cortical Analyses

The Brain Anatomical Analysis using Diffeomorphic deformation (BAAD 4.31—http://www.shiga-med.ac.jp/~hqbioph/BAAD/Welcome_to_BAAD.html) (31) and Statistical Parametric Mapping tool (SPM12, https://www.fil.ion.ucl.ac.uk/spm/software/spm12/) were used to calculate the cortical alteration patterns. This tool includes a Computational Anatomy Toolbox (CAT12)-based (http://www.neuro.uni-jena.de/cat/), T1-weighted-3D diffeomorphic segmentation after inhomogeneity correction, T1-weighted-3D quality check assessment, and Total Intracranial Volume (TIV) estimation. T2-weighted-3D was included in the multimodal segmentation to correct brain atrophy estimation for the presence of white matter lesions. Brain Anatomical Analysis using Diffeomorphic deformation integrates CAT12 tool for the normalization to the standard MNI space (31). Moreover, it provides at subject level a voxel-wise non-parametric statistical map of GM and white matter alterations, comparing each participant's normalized and segmented brain MRI images to the age- and sex-matched normative data with the SnPM13 tool (http://warwick.ac.uk/snpm), as previously described (31). See first level maps comparison, PD-MCI subgroups vs. healthy population, in Supplementary Figure 1; in addition, further methodological details are reported in our previous work (18).

Statistical maps of GM alterations were then included in a second level GLM analysis comparing PD-MCI-Aβ+ vs. PD-MCI-Aβ- subgroups to assess for possible GM differences. Areas of shared alterations were tested with a conjunction analysis including the two subgroups as factors. To reduce false positives (32), a Bayesian Probabilistic Threshold-Free Cluster Enhancement (TFCE) method was used to define GM patterns differences in PD-MCI subgroups. A significant threshold family-wise error (FWE) corrected of *p* < 0.001 was adopted. The atlas (AAL3v1, http://www.gin.cnrs.fr/en/tools/aal/) provided with the automated anatomical parcellation tool was used for results.

### Statistical Analyses

Pairwise differences between the two subgroups (PD-MCI-Aβ+ vs. PD-MCI-Aβ-) in demographic, motor, clinical, cognitive, and behavioral characteristics were assessed with Mann–Whitney U test or Fisher's exact test for categorical variables. Each cognitive raw score was converted to z-score using the Italian normative data, considering as pathologic a performance below the −1.5 SD cut-off. The z-compound (mean z-score among tests of each cognitive domain) was also calculated. Moreover, we tested the hypothesis that increased amyloid load might alter a cognitive domain performance, by running a univariate linear regression analysis between the significantly affected cognitive domains and the mean SUVR, extracted from each cluster/subcortical region with a marked amyloid deposition. Statistical analyses were run with “R software” (R version 3.6.2 (2019-12-12)—Copyright (C) 2019).

## Results

### Clinical Characteristics

Seventeen out of 25 PD-MCI patients were classified as Aβ- and 8 Aβ+. The two groups did not differ in demographic, clinical, motor, and behavioral variables including global cognition scales scores (MMSE and MoCA) (see Table 1).

**Table 1:** Demographical, clinical, motor, and behavioral characteristics of PD-MCI Aβ+ vs. Aβ-.

		**PD-MCI Aβ+**	**PD-MCI Aβ−**	**Mann-Whitney U Test/Fisher Test**
		**Mean (SD)/Frequency**	**Mean (SD)/Frequency**	
Demographics	Sex (male/female)	7/1	9/8	0.182
	Age (years)	72.75 (3.69)	68.82 (7.36)	0.074
	Education (years)	11.25 (3.95)	8.47 (3.60)	0.111
Clinical characteristics	Disease Duration (years)	8.63 (2.61)	10.58 (5.78)	0.578
	Age at motor symptoms' onset (years)	64.75 (4.26)	58.76 (9.42)	0.091
	LEDD (mg tot/die)	886.875 (405.80)	887.88 (429.60)	1.000
	DAED (mg tot/die)	115 (125.47)	124.27 (123.62)	0.816
Motor characteristics	MDS-UPDRS I	11	15.29 (10.77)	1.000
	MDS-UPDRS II	10	19.43 (7.76)	0.250
	MDS-UPDRS III	27.33 (15.70)	29.4 (15.27)	0.815
	MDS-UPDRS IV—fluctuation	2.4 (2.88)	3.91 (3.78)	0.600
	MDS-UPDRS IV—dyskinesia	0	1.18 (2.13)	0.245
	MDS-UPDRS total score	34	65.43 (23.07)	0.250
	H&Y	2.58 (0.91)	2.37 (1.00)	0.837
Functional independence and global cognitive status	ADL	5.4 (0.51)	5.52 (0.62)	0.447
	IADL	4.25 (1.40)	4.82 (2.30)	0.406
	PD-CFRS	5.875 (4.48)	7.31 (5.50)	0.538
	MMSE (corrected score)	24.71 (1.84)	24.67 (1.81)	0.884
	MoCA (corrected score)	19.18 (2.00)	20.31 (2.57)	0.391
Behavioral measures	PDQ-8	8.57 (7.63)	11 (5.81)	0.270
	APATHY	14.57 (2.44)	18.77 (6.44)	0.130
	STAI-Y1	35.25 (5.70)	41.13 (9.07)	0.154
	STAI-Y2	39.71 (10.24)	45.53 (8.60)	0.157
	BDI-II	11.00 (6.34)	9.93 (6.89)	0.657
	BIS-11	60.80 (10.60)	65.78 (10.43)	0.479
	QUIP-RS	8.14 (7.73)	6.33 (8.52)	0.590

*Mann–Whitney U test was run to test between-group differences. Fisher Exact test was used for the sex variable. ADL, activities of daily living; BDI-II, Beck Depression Inventory-II; BIS-11, Barratt Impulsiveness Scale; DAED, dopamine agonist equivalent dose; HC, healthy controls; IADL, Instrumental activities of daily living; LEDD, levodopa equivalent daily dose; MDS-UPDRS, Movement Disorder Society Unified Parkinson's Disease Rating Scale; MMSE, Mini-Mental State Examination; MoCA, Montreal Cognitive Assessment; PD-CFRS, Parkinson's Disease -Cognitive Functional Rating Scale; PDQ-8, Parkinson's Disease Questionnaire; QUIP-RS, Questionnaire for Impulsive-Compulsive Disorders in Parkinson's Disease–Rating Scale; STAI (Y-1, Y-2), State-Trait Anxiety Inventory*.

Looking at the performances in each single cognitive test, the two cohorts showed no differences in any individual test of the five investigated cognitive domains. However, PD-MCI-Aβ+ showed worse performance (z-compound) in the overall executive domain than PD-MCI-Aβ- (*p* = 0.037, see Table 2).

**Table 2:** Comparison of the neuropsychological scores between PD-MCI subgroups (Aβ+ vs. Aβ-).

		**PD-MCI Aβ+**		**PD-MCI Aβ−**		**Mann Whitney *U* Test**
**Cognitive domains**	**Cognitive tests**	**Median**	**IQR 25–75**	**Median**	**IQR 25–75**	***p*-values**
Attention/working memory	TMT A	42.50	35 to 69.8	72.50	54.3 to 91	0.076
	TMT B	355	229 to 367	248	198.5 to 330.3	0.542
	TMT B-A	314.50	188 to 336	174.50	130.5 to 238.3	0.553
	DSS (WAIS-IV)	8	8 to 10.5	9.50	8 to 11	0.635
	**z-compound**	−0.22	−0.6 to 0.1	−0.63	−1.4 to 0.1	0.124
Executive domain	Phonemic fluency	30.50	27.5 to 33.5	30	23 to 35	0.944
	Stroop/color task-Time	48.12	14.2 to 73	35.80	21.1 to 47.8	0.759
	Stroop/color task-Errors	4.40	1.7 to 8.1	0.38	0 to 2.3	0.092
	Similarities (WAIS-IV)	8	7.5 to 9	7.50	6.8 to 10	0.761
	CDT	11	10.5 to 12	12	10 to 13	0.797
	**z-compound**	−1.48	−1.9 to −1.1	−0.60	−1 to −0.5	**0.037**
Memory domain	Prose memory test	6	5 to 6.5	4.50	1.8 to 7.3	0.545
	Prose memory test delayed	8	5 to 10	6.50	4.5 to 9.25	0.500
	ROCF delayed	8.40	6.8 to 11.8	9.9	7.4 to 11.4	0.806
	WPAT	11.50	11.3 to 16.3	10.50	8 to 11.6	0.105
	**z-compound**	−1.07	−1.3 to 0.8	−1.60	−1.9 to −0.9	0.140
Visuospatial domain	ROCF copy	19.40	12.6 to 25	24.50	20.8 to 28.7	0.178
	VOSP—incomplete letters subtask	16.50	13 to 18	17	15 to 18	0.747
	Benton—JLO	18	16 to 22.5	19	9.5 to 22	0.806
	**z-compound**	−2.80	−3.4 to −2	−2.21	−2.6 to −1.6	0.344
Language domain	Category fluency	30	30 to 34.5	31	28 to 39	0.679
	Naming Task	28.30	27 to 29.2	29.50	28.4 to 30.4	0.211
	**z-compound**	−0.83	−1.1 to −0.6	−0.94	−1.5 to −0.3	1.000
Apraxia	Apraxia	19.25	19 to 20	19	17.8 to 19.8	0.253

*IQR, interquartile range; WAIS IV, Wechsler Adult Intelligence Scale—Fourth Edition; VOSP, Visual Object and Space Perception; TMT, Trail Making Test; WPAT, Word paired associated task; JLO, Judgement of Line Orientation; ROCF, Rey-Osterrieth complex figure test; CDT, Clock drawing task; DSS, Digit Span Sequencing. Statistically significant results are in bold type*.

### Cortical and Subcortical β-Amyloid Distribution in PD-MCI-Aβ+

**Figure 1:**
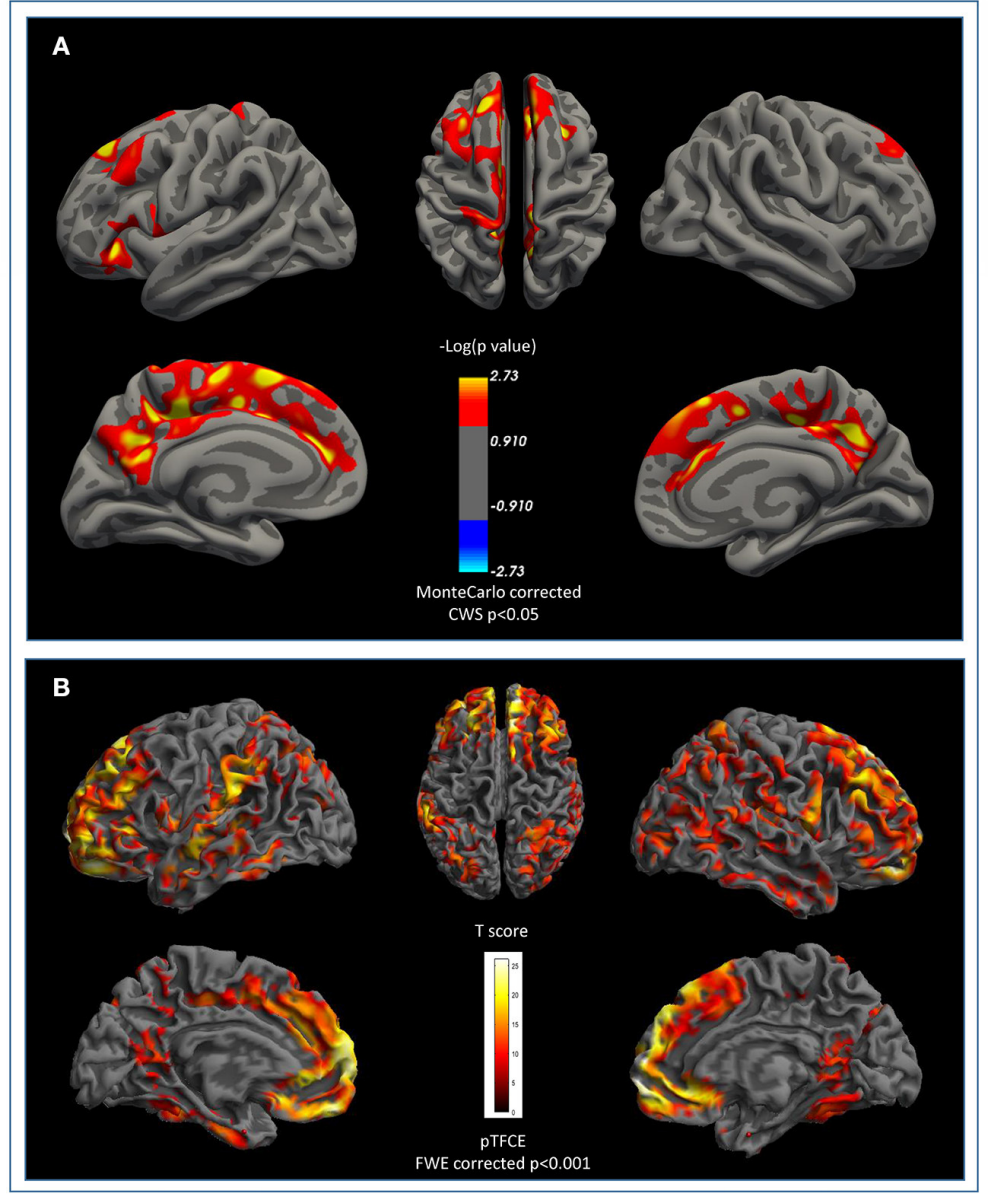
β-Amyloid cortical deposition and brain atrophy in PD-MCI. **(A)** Surface based comparison between standardized uptake value ratios (SUVR) in PD-MCI-Aβ+ vs. Aβ-. Areas that survived a cluster-wise Monte-Carlo correction (*p* < 0.05) adjusted for two-hemispheres are displayed. Cerebellum cortex was used as reference region for partial volume correction. **(B)** Shared pattern of atrophy between PD-MCI-Aβ+ vs. Aβ- compared to healthy matched population at FWE pTFCE *p* < 0.001. No areas survived after β-amyloid +/– at TFCE uncorrected *p* < 0.001 threshold. FWE, family-wise error; TFCE, Threshold-Free Cluster Enhancement.

Cortical surface analysis showed significant (cluster-wise Monte Carlo corrected) bilateral amyloid depositions in the caudal and rostral middle frontal cortices, in the right precuneus and in left paracentral and pars triangularis areas, in PD-MCI-Aβ+ compared with Aβ- (see Figure 1A and Table 3). Given that values of FMM SUVR in subcortical regions (nucleus accumbens, caudate, putamen, amygdala, pallidum, thalamus, and hippocampus) were highly correlated between hemispheres (*p* < 0.0001), we analyzed the right-left averaged ROIs scores. The only subcortical region showing significant amyloid deposition was the putamen (*p* = 0.038), despite this result was no more significant following multiple comparisons correction (see Table 4).

**Table 3:** Pattern of β-amyloid cortical deposition in PD-MCI.

	**Regions**	**MNI**	**MNI**	**MNI**	**Cluster-wise MC**	**N of vertices**	**Size**	**Max –**
		**X**	**Y**	**Z**	***p*-value**		**(mm^**2**^)**	**log10(*p*)**
Rh	Caudal middle frontal gyrus	26.5	18	41.8	0.0001	4,702	2429.8	3.408
	Precuneus	11	−55.7	38.4	0.0001	5,647	2383.4	3.625
Lh	Paracentral lobule	−12.9	−28.6	47.2	0.0001	15,206	7136.8	4.962
	Pars triangularis	−38.7	30.4	−6.7	0.0001	4,339	2041.4	3.301
	Rostral middle frontal gyrus	−30.4	26.1	38.6	0.0031	2,577	1494.8	2.951

*Cortical surface-based comparison between SUVR in PD-MCI β-Amyloid +/–. Areas that survived a cluster-wise p < 0.05 Monte-Carlo corrected threshold adjusted for two-hemispheres values. Cerebellum cortex was used as reference region after partial volume correction in PETsurfer. Rh, right hemisphere; Lh, left hemisphere; MC, Monte Carlo correction. Coordinates were displayed in MNI space*.

**Table 4:** Pattern of β-amyloid striatal and extra-striatal subcortical deposition.

	**PD-MCI Aβ**– **(*****n*** **= 16)**		**PD-MCI Aβ+** **(*****n*** **= 7)**		**Mann Whitney U test**
**Subcortical region (average L/R SUVR)**	**Median**	**2.5 to 97.5 P**	**Median**	**2.5 to 97.5 P**	***p-value*** ***(uncorrected)***
Nucleus accumbens	−1.14	−3.32 to 1.72	−0.39	−2.63 to 1.51	0.423
Putamen	1.15	0.45 to 2.78	1.73	1.29 to 3.14	**0.038**
Caudate	0.38	0.08 to 3.03	0.98	0.56 to 2.14	0.161
Hippocampus	1.13	0.76 to 1.84	1.06	0.88 to 1.41	0.689
Globus pallidus	2.17	0.59 to 3.51	2.21	0.51 to 2.47	0.738
Amygdala	1.21	0.67 to 2.40	1.34	0.96 to 1.62	0.345
Thalamus	1.38	−0.06 to 1.89	1.7	0.32 to 2.16	0.204

*Significant result (in bold) was not corrected for multiple comparison testing, following this correction, this difference was not statistically significant. Standardized uptake value ratios (SUVR); L/H, left-right; P, percentiles; PD-MCI, Parkinson's disease with mild cognitive impairment; Aβ, β-amyloid positive vs. negative*.

### Cortical Gray Matter in PD-MCI-Aβ+

Cortical voxel-based morphometry (VBM) analysis showed a similar pattern of brain atrophy in PD-MCIAβ+ and PD-MCIAβ-. Conjunction analysis showed that PD-MCI subgroups shared a similar pattern of atrophy in bilateral orbitofrontal, middle frontal, superior middle frontal regions, rostral and middle cingulate cortex, and fusiform regions and orbicular and superior temporal region on the right hemisphere (see Figure 1B and Table 5).

**Table 5:** Areas of gray matter atrophy in PD-MCI vs. controls.

**AAL3 atlas**				**Cluster**	**Cluster**	**Peak**	**Peak**	**Peak**
	**MNI** **(X)**	**MNI** **(Y)**	**MNI** **(Z)**	**N voxels**	**P** **(FWE-corr)**	**P** **(FWE-corr)**	**T**	**Z**
Medial OFC-L	−9	51	−21	64,435	0.0000	0.0000	Inf	Inf
Medial OFC-R	13.5	52.5	−18			0.0000	Inf	Inf
Medial OFC-R	−15	24	−16.5			0.0000	Inf	Inf
Frontal superior L	−26	38	44			0.0000	17.05	7.21
Frontal superior medial L	8	42	53			0.0000	14.19	6.75
Fusiform R	45	−37.5	−16.5	6,815	0.0000	0.0000	19.0372	7.4730
Fusiform R	40.5	−45	−15			0.0000	15.0621	6.9023
Temporal inferior R	54	−24	−21			0.0000	14.8525	6.8672
Precuenus R	7.5	−64.5	64.5	11	0.0001	0.0001	11.1572	6.1303
Precuenus L	−4.5	−49.5	42	392	0.0000	0.0001	10.8696	6.0611
Precuenus L	−6	−49.5	55.5			0.0002	10.6720	6.0124
Cingulate middle–L	−12	−39	36			0.0002	10.6214	5.9998
Precuenus R	16.5	−66	28.5	243	0.0000	0.0002	10.5749	5.9881
Fusiform R	34.5	−7.5	−36	29	0.0000	0.0006	9.7211	5.7626
Caudate L	−9	13.5	3	21	0.0000	0.0008	9.5086	5.7029

### Association Between β-Amyloid Load and Cognitive Functions

**Figure 2:**
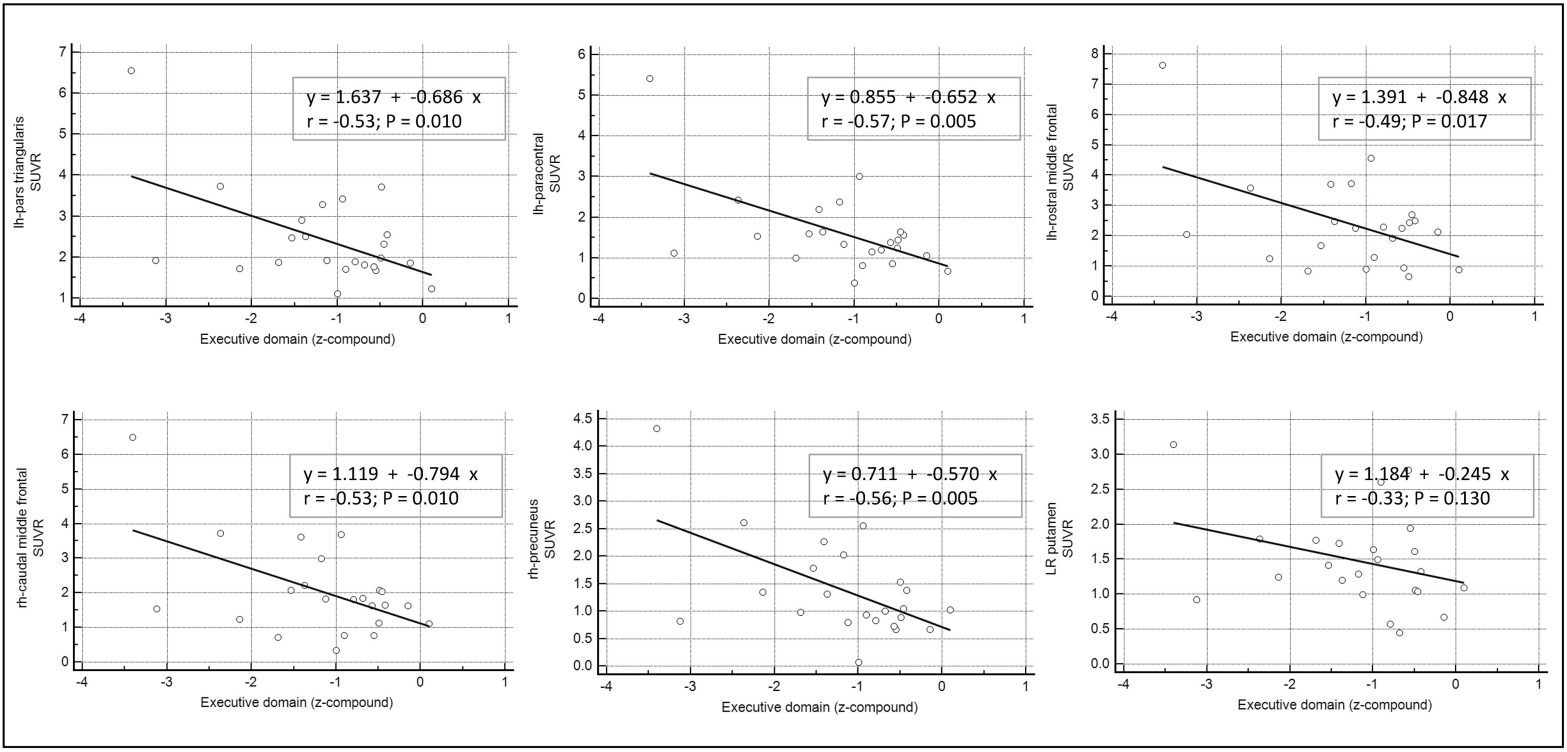
Correlation between β-amyloid load in cortical and subcortical areas and cognitive performance. Univariate linear regression model between the executive domain (*z*-compound) and the mean standardized uptake value ratios (SUVR) of each significant cortical and subcortical ROIs.

Only the executive domain was affected by the amyloid presence (see Table 2), and therefore only this domain was considered for further analyses. There was a negative linear relationship between cortical regions with significant amyloid load and worsening in executive performance. Namely, we found a significant association with the following regions: in the right hemisphere, the caudal middle frontal gyrus (*r* = −0.53; p = 0.01), and the precuneus (*r* = −0.56; *p* = 0.005); while in left hemisphere, the rostral middle frontal (*r* = −0.49; *p* = 0.017), the paracentral (*r* = −0.57; *p* = 0.005), and the pars triangularis regions (*r* = −0.53; *p* = 0.01). By contrast, the subcortical L/R-putamen did not show any significant correlation with the executive domain dysfunctions (*r* = −0.33; *p* = 0.130) (see Figure 2).

## Discussion

In the present study, we used simultaneous PET/MRI imaging study to investigate how Aβ burden affects cognitive performance in PD-MCI patients, diagnosed with a comprehensive neuropsychological assessment, allowing a level II diagnosis.

We found that in our PD-MCI cohort, 8/25 (31%) patients were Aβ+, corroborating previous data on brain amyloid prevalence in non-demented PD (20). While all demographic, clinical, behavioral, and motor variables did not differ, PD-MCI-Aβ+ showed a trend for higher age of motor's symptoms onset, suggesting that extracellular β-amyloid deposition is an age-related pathological marker (33).

From a neuropsychological point of view, PET amyloid positivity was associated with worse performance in the executive domain, supporting previous PD literature on the contribution of Aβ on attentive and executive dysfunctions (10, 34, 35).

Interestingly, brain Aβ did not impact other cognitive domains such as visuo-spatial and semantic memory, which are considered highly sensitive in detecting the deterioration and progression to severe impairment and dementia (1, 2, 18, 23, 36–38). These results seem to suggest that PD-MCI-Aβ+ cognitive pattern does not share the typical cognitive profile of MCI due to Alzheimer's disease (AD).

The observation of a significant proportion of PD-MCI-Aβ- can possibly suggest that Aβ is not the main pathogenetic cause of cognitive deterioration in PD (10, 11, 18, 34). Instead, amyloidosis may enhance early deficits in those cognitive domains already vulnerable to PD primary pathology (2, 18), possibly indicating that in PD concomitant conditions may aggravate cognition and herald dementia (39–41). In this regard, in a recent longitudinal study performed in a large PD *de novo* cohort, we concluded that amyloid burden together with asymmetric dopaminergic loss (in the left hemisphere) and aging exhibited independent and interactive contributions to PD-cognitive progression (namely, involving attentive/executive alterations) (42).

One of the strengths of our study is the quantification of cortical Aβ load using a surface-based approach and SUVR measures, not reported in our previous work (18).

We found increased Aβ uptake in the cortical areas, mostly located in the frontal (i.e., caudal and rostral middle frontal cortices and pars triangularis) as well as in the parietal regions (i.e., paracentral lobule and precuneus), which overall correlated with a poor executive performance.

From a cognitive perspective, the involvement of these areas as well as their key role within the fronto-striatal circuit is well-established. They subserve a wide range of cognitive tasks, such as executive functions and working-memory abilities, involving information monitoring and manipulation, planning and organization of complex behaviors, and attention shifting (43), with frontal striatal network alterations underlying early changes in PD, and predicting cognitive decline as well as dementia (44, 45). Noteworthy, the present findings corroborate our previous PET-amyloid evidence in a PD *de novo* cohort (10), showing that increased cortical and subcortical amyloid was associated with a worse performance in attentive/executive domains. Yet, this reinforces the concept of cortical and subcortical amyloid accumulation as an additional biomarker contributing to cognitive impairment in PD (10, 12, 46).

Increased FMM uptake in PD-MCI-Aβ+ was also present in the right precuneus, which is anatomically and functionally connected with subcortical striatal areas and implied in functional abnormalities in PD-MCI (47). Our findings support previous PET imaging studies in non-demented PD, despite the fact that different Aβ-tracers were used (46, 48), showing a greater amyloid burden particularly in the precuneus and frontal regions, as well as their inverse correlation with cognitive performance (46). Other evidence did not corroborate this pattern, reporting no differences between PD-MCI and PD with normal cognition, possibly due to the small sample size (34). Evidence of amyloid deposition in the parietal regions seems to be consistent across PET imaging studies (12, 48, 49). Of note, one reported an association with visuospatial impairments (49) but no significant correlations between amyloid and executive functions; however, the entire Lewy bodies spectrum was analyzed.

Overall, previous studies in PD population yielded mixed results, and as highlighted in the Petrous et al. review (13), there is only a few published evidence about the role of Aβ in PD-MCI, due to the heterogeneous nature of this clinical syndrome as well as the high variability in the MCI-assessment between studies.

Further, PD-MCI-Aβ+ presented increased FBB uptake in the putamen, but without significant correlation with executive functions. In a previous analysis of the PPMI dataset, we had reported a correlation between executive tests and putamen Aβ accumulation, but this included only early *de novo* PD with limited cognitive characterization, not allowing to diagnose MCI (10). Another study reported a correlation but using a different PET tracer and in a cohort with various cognitive alterations (50). It is possible that lack of correlation in our cohort was related to the limited number of PD-MCI-Aβ+, but future studies should focus on this specific marker of PD-related amyloidosis exploring also its relevance on the magnitude of levodopa response and on motor complications.

Finally, we also evaluated GM volumetric changes at MRI. Results showed similar atrophy patterns in right fronto-temporal regions in both PD-MCI subgroups, replicating previous findings adopting different structural analysis methods (5, 7). Interestingly, cortical VBM patterns were similar in PD-MCI with and without Aβ deposition, and excluded the presence of middle-temporal atrophy, a characteristic of early AD (51). This supports our previous findings of absence of AD-atrophy pattern in the LBD patients with dementia (18).

Our current work has limitations. Each subgroup (Aβ+ and Aβ-) included a relatively small number of PD-MCI subjects, resulting in lower statistical power to detect differences. However, results are aligned with previous evidence (18) of an independent role of Aβ in shaping MCI-profile and accelerating cognitive deficits in PD. Moreover, regarding the neuroimaging analyses we acknowledge that the small sample size can possibly affect the results; however, to reduce the false positive, we adopted TFCE or Monte Carlo FWE corrections, while to reduce the false negative an explorative uncorrected *p* < 0.001 was adopted.

Greater numerosity is needed to further explore the relationship between Aβ deposition and increased dementia-risk profiles. In particular, it would be worth exploring if low concentration of Aβ, below AD-range thresholds, may also have clinical relevance (13) in presence of an ongoing multisystem neurodegenerative process like PD. In this regard, a further limitation is that we did not screen our PD-MCI for APOE genotype which is now considered the main risk factor for cognitive decline in elderly patients with PD and Dementia with Lewy Bodies (34, 52, 53).

Finally, we did not include a control group of healthy controls and PD without cognitive deficits to assess age-related brain amyloid distribution. However, a recent publication of elderly individuals without cognitive deficits demonstrated areas of cerebral amyloid deposition (i.e., prefrontal, precuneus) similar to those observed in our Aβ+PD-MCI patients, suggesting that PD-related amyloidosis somehow overlaps age-related amyloid accumulation (54).

In conclusion, our study suggests that amyloid burden in the fronto-striatal network may play a role in worsening executive abilities in PD-MCI patients. Furthermore, the observation of more than 60% Aβ- in our PD-MCI patients led us to speculate about the independent role of amyloid load for PD-MCI. Notably, amyloid accumulation in our cohort was not associated with typical AD cognitive and brain atrophy pattern nor with specific clinical features and demographic characteristics, highlighting its potential unspecific contribution. Considering that up to 80% of PD patients may ultimately develop dementia with negative consequences on life quality and expectancy, a better understanding of the involved predictors is of key importance. We believe clarifying Aβ role in cognitive impairment and progression is clinically relevant, especially in the context of emerging applicability of amyloid-related treatments.

## Data Availability Statement

The original contributions presented in the study are included in the article/Supplementary Material, further inquiries can be directed to the corresponding author.

## Ethics Statement

The studies involving human participants were reviewed and approved by Azienda Ospedale Università di Padova. The patients/participants provided their written informed consent to participate in this study.

## Author Contributions

AA and RB contributed to conception and design of the study. FP and MG organized the database. LW performed the statistical analysis and wrote sections of the manuscript. MG wrote the first draft of the manuscript. All authors contributed to manuscript revision, read, and approved the submitted version.

## Funding

We are grateful to GE-Healthcare for the liberal contribution of all doses of [^18^F]flutemetamol used in the present work and for covering publication fees.

## Conflict of Interest

AA has received compensation for consultancy and speaker related activities from UCB, Boehringer Ingelheim, General Electric, Britannia, AbbVie, Kyowa Kirin, Zambon, Bial, Theravance Biopharma, Roche, Medscape, Ever Pharma; he receives research support from Bial, Lundbeck, Roche, Angelini Pharmaceuticals, Horizon 2020—Grant 825785, Horizon2020 Grant 101016902, Ministry of Education University and Research (MIUR) Grant ARS01_01081, Cariparo Foundation. He serves as consultant for Boehringer–Ingelheim for legal cases on pathological gambling. The remaining authors declare that the research was conducted in the absence of any commercial or financial relationships that could be construed as a potential conflict of interest.

## Publisher's Note

All claims expressed in this article are solely those of the authors and do not necessarily represent those of their affiliated organizations, or those of the publisher, the editors and the reviewers. Any product that may be evaluated in this article, or claim that may be made by its manufacturer, is not guaranteed or endorsed by the publisher.

## Supplementary Material

Supplementary Figure 1 Cortical atrophy in PD-MCI subgroups as compared to healthy population. Nonparametric comparison of PD-MCI gray matter atrophy compared to age and sex matched normal control (NC). A TFCE *p* < 0.05 FWE corrected threshold was used. TFCE, threshold-free cluster enhancement; FWE, family-wise error.
