# Supplementary figures and images for: Quantification of Brain β-Amyloid Load in Parkinson's Disease With Mild Cognitive Impairment: A PET/MRI Study
Source: Front Neurol. 2022 Mar 1;12:760518. doi: 10.3389/fneur.2021.760518 (PMC8921107; doi:10.3389/fneur.2021.760518)

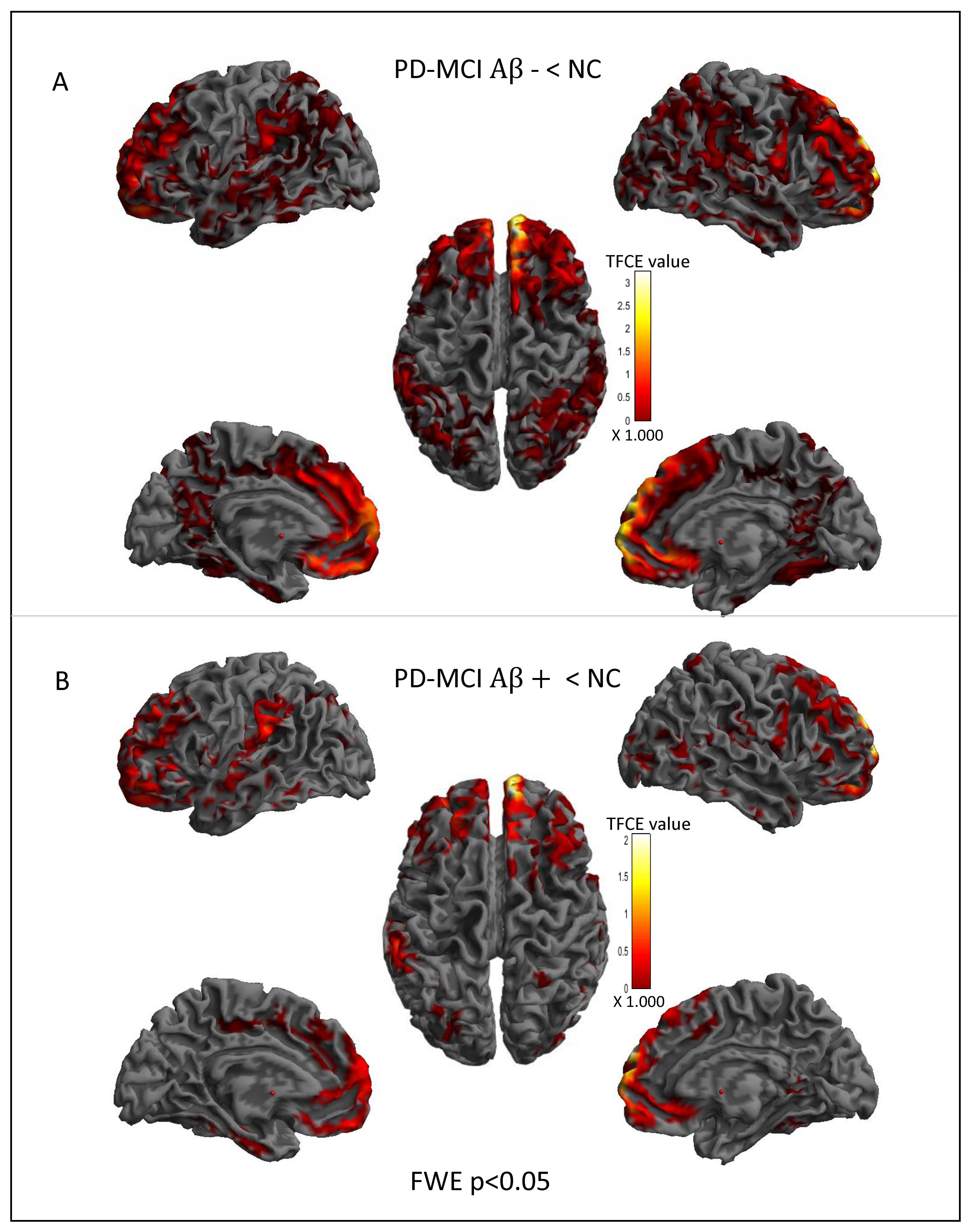

Supplement: Supplementary Figure 1 — Cortical atrophy in PD-MCI subgroups as compared to healthy population. Nonparametric comparison of PD-MCI gray matter atrophy compared to age and sex matched normal control (NC). A TFCE p < 0.05 FWE corrected threshold was used. TFCE, threshold-free cluster enhancement; FWE, family-wise error. [file Image_1.TIFF]
